# Stock Market Forecasting Using Restricted Gene Expression Programming

**DOI:** 10.1155/2019/7198962

**Published:** 2019-02-05

**Authors:** Bin Yang, Wei Zhang, Haifeng Wang

**Affiliations:** School of Information Science and Engineering, Zaozhuang University, Zaozhuang, China

## Abstract

Stock index prediction is considered as a difficult task in the past decade. In order to predict stock index accurately, this paper proposes a novel prediction method based on S-system model. Restricted gene expression programming (RGEP) is proposed to encode and optimize the structure of the S-system. A hybrid intelligent algorithm based on brain storm optimization (BSO) and particle swarm optimization (PSO) is proposed to optimize the parameters of the S-system model. Five real stock market prices such as Dow Jones Index, Hang Seng Index, NASDAQ Index, Shanghai Stock Exchange Composite Index, and SZSE Component Index are collected to validate the performance of our proposed method. Experiment results reveal that our method could perform better than deep recurrent neural network (DRNN), flexible neural tree (FNT), radial basis function (RBF), backpropagation (BP) neural network, and ARIMA for 1-week-ahead and 1-month-ahead stock prediction problems. And our proposed hybrid intelligent algorithm has faster convergence than PSO and BSO.

## 1. Introduction

Stock market plays a leading and crucial role in the market mechanism, which connects the savers and investors [1, 2]. The operating mechanism of the stock market reflects the situation of national economy and is recognized as the signal system of the national economy [3, 4]. Because of some uncontrollable factors, such as economic growth, economic cycle, interest rate, fiscal revenue and expenditure, money supply, and price, the prediction of the stock market index is considered to be a difficult job [5–7].

Many machine learning (ML) methods containing statistical models, artificial neural networks, and hybrid prediction models have been proposed to model and predict the stock index. As a classical statistical model, the ARIMA model has proposed to predict the New York Stock Exchange (NYSE) and Nigeria Stock Exchange (NSE), and the results revealed that the ARIMA model performed better for short-term prediction [8–10]. Compared with the ARIMA model, the artificial neural network (ANN) model has more strong prediction and modeling ability. Adebiyi et al. made the comparison of ARIMA and ANN models for stock price prediction and found that the stock forecasting model based on ANN approach had superior performance over ARIMA models [11].

In the past decades, many ANN models have been employed for solving real problems, especially stock market prices forecasting [12, 13]. Dong et al. presented backpropagation (BP) neural networks for stock prediction [14]. Feedforward ANN was proposed to predict price movement of the stock market [15]. Akita et al. proposed a novel deep learning method based on paragraph vector and long short-term memory (LSTM) to predict the Tokyo Stock Exchange [16]. Rout et al. used the radial basis function (RBF) neural network to forecast DJIA and S&P 500 stock indices [17]. Wang et al. proposed a novel method based on complex-valued neural network (CVNN) and Cuckoo search (CS) algorithm to forecast stock price [18]. Chen et al. presented the flexible neural tree (FNT) ensemble technique to analyze 7-year Nasdaq-100 main index values and 4-year NIFTY index values [19].

However, the existing methods mainly trained the black box with the training sample. The model could change its internal structure and parameters to make it approximate to the training sample. The gained model could not display the distinct input-output relationship and deeply understand the internal mechanisms of real-world problems. And, in most of these methods, all variables are input into the models, which easily lead to overfitting problem. Recently the methods based on mathematical formulations have been proposed to predict time series, which could clearly indicate the mathematical relationship between the input data and output data. Zuo et al. proposed that gene expression programming (GEP) was utilized to identify differential equation for time series prediction [20]. Graff et al. proposed genetic programming (GP) to forecast time series [21]. Grigioni et al. proposed a modified power-law mathematical model to predict the blood damage sustained by red cells with the load history [22]. Mina et al. proposed a beta-function formula to forecast the maxillary arch form [23]. Chen et al. identified ordinary differential equations (ODEs) to forecast the small time scale traffic measurements data and proved that the ODE model was more feasible and efficient than ANN models [24].

As a classical nonlinear differential equation, the S-system model has been proposed to predict time series and identify genetic networks. Zhang and Yang proposed a restricted additive tree (RAT) to represent the S-system model for stock market index forecasting [25]. However, the RAT method has nonlinear structure and is implemented inconveniently. In this paper, a novel stock index prediction method based on S-system model is proposed. Restricted gene expression programming (RGEP) is proposed to encode and optimize the structure of S-system. In order to optimize the parameters of the S-system model accurately, a new hybrid intelligent algorithm based on the brain storm optimization (BSO) algorithm and particle swarm optimization (PSO) algorithm is proposed.

Dow Jones Index, Hang Seng Index, and NASDAQ Index are old and famous stock indexes in the world, which are usually utilized to reflect the development of the global economy. Shanghai Stock Exchange Composite Index and SZSE Component Index represent the general trend of China's stock market and economic development. These five stock indexes have been considered as the standard datasets to evaluate the performance of stock prediction models [26–30]. Thus, Dow Jones Index, Hang Seng Index, NASDAQ Index, Shanghai Stock Exchange Composite Index, and SZSE Component Index are collected to validate the performance of our proposed method.

## 2. Background Concepts and Related Technologies

### 2.1. Data Description

**Figure 1:**
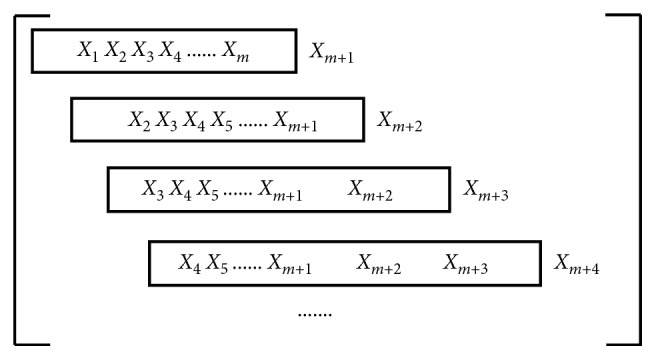
Data structure.

Let stock time series data to be [*X*_1_, *X*_2_,…, *X*_*T*_] (*T* is the number of time points). Generally, the data from the past time points are used to predict the data at the current time point.  Figure 1 shows an example of data partition with *m* input variables. The data in the box are utilized as the input vector, and the data on the right side of the box is the prediction value. Two forecasting strategies, 1-week-ahead (*m*=7) and 1-month-ahead (*m*=30), are utilized in this paper.

### 2.2. S-System Model

The S-system model has a complex and powerful structure, which captures the dynamic nature of the real system, and achieves a good performance in the terms of precision and flexibility [31, 32]. The *i*th nonlinear differential equation in S-system is described as follows:

(1) $$\begin{array}{c} \frac{dX_{i}}{dt}=\alpha_{i}\overset{N}{\underset{j=1}{\prod }}X_{j}^{g_{ij}}-\beta_{i}\overset{N}{\underset{j=1}{\prod }}X_{j}^{h_{ij}}, \end{array}$$

where *N* is the number of equations, *X*_*i*_ is the *i*th variable, *α*_*i*_ and *β*_*i*_ are the rate constants of production function and consumption function, and *g*_*ij*_ and *h*_*ij*_ are the kinetic orders.

### 2.3. Brain Storm Optimization Algorithm

Brain storm optimization (BSO) algorithm is a new swarm intelligence optimization algorithm, which was proposed by Shi in the year 2011 [33]. In BSO, the cluster algorithm is proposed to search the local optimal solution and the global optimal solution is obtained through the comparison of all local optimal solutions. Mutation strategy is utilized to enhance the diversity of the algorithm and avoid obtaining local optimal solution [34]. The BSO process is described as follows:

1. Initialize the population and generate *N* potential solutions (*x*_1_, *x*_2_,…, *x*_*N*_).
2. The *k*-means clustering algorithm is utilized to divide the *N* individuals into *k* classes. The fitness value of each individual is calculated. The best individual in each category is selected as the central individual.
3. Select randomly the central individual of a class and mutate it with a random disturbance.
4. Update the individual with the following four methods.
  - Select randomly a class (the probability is proportional to the number of individuals in each class). A new individual (*x*_s_′) is generated by adding the random perturbation to the central individual (*x*_s_), which is defined as follows:

(2) $$\begin{array}{c} x_{\text{s}}^{\prime }=x_{\text{s}}+\zeta \times N\left(\mu ,\sigma \right), \end{array}$$

where *N*(*μ*, *σ*) is the Gaussian random function and *ζ* is the factor that balances the random number, which is defined as follows:

(3) $$\begin{array}{c} \zeta =\log\text{ sig}\left(\frac{\left(0.5\;*\text{ max\_iteration}-\text{current\_iteration}\right)}{k}\right)*\text{ rand}\left(\right), \end{array}$$

where log sig is a logarithmic *S-*transform function, max_interation is the maximum number of iterations in the algorithm, current_interation is the number of current iterations, is the gradient which is utilized to control the logarithmic *S*-transformation function, and rand() is the random number in the interval [0, 1].

(b) Randomly select a class and an individual in the selected class. A new individual is created with the selected individual and Gaussian value by equations (2) and (3).
(c) Select randomly two classes, and two central individuals from the two classes are utilized as the candidate individuals *x*_s1_ and *x*_s2_, which are fused with the following formula:
  (4) $$\begin{array}{c} x_{\text{s}}=\lambda \times x_{\text{s}1}+\left(1-\lambda \right)\times x_{\text{s}2}, \end{array}$$
  where *λ* is a random number in the interval [0, 1].

After merging the candidate individuals, the individual is updated according to the formula (2).

(d) Two candidate individuals *x*_s1_ and *x*_s2_ are selected randomly from the two selected classes. The fusion and updating operators are implemented with equations (2) and (4).

After the new individual is generated, its fitness value is calculated. Compared with the fitness values of the candidate individuals, the individuals with the better fitness values are selected to the next generation. When *N* new individuals are generated, enter the next iteration process.

(5) When the maximum iteration number is reached, algorithm stops; otherwise, go to step (2).

### 2.4. Particle Swarm Optimization Algorithm

The particle swarm optimization (PSO) algorithm is a classical swarm intelligent method [35]. In PSO, each potential solution is presented by a particle. A swarm of particles [*x*_1_, *x*_2_,…, *x*_*N*_] moves in order to search the food source, with the moving velocity vector [*v*_1_, *v*_2_,…, *v*_*N*_]. At each step, each particle searches the optimal position separately in the space, which is recorded in a vector *P*_best_*i*__. The global optimal position is searched among all the particles, which is kept as *G*_best_ [36].

At each step, a new velocity for the particle *i* is updated by the following equation:

(5) $$\begin{array}{c} v_{i}\left(t+1\right)=w\;*\;v_{i}\left(t\right)+c_{1}r_{1}\left(P_{{\text{best}}_{i}}-x_{i}\left(t\right)\right)+c_{2}r_{2}\left(G_{\text{best}}\left(t\right)-x_{i}\left(t\right)\right), \end{array}$$

where *w* is the inertia weight and impacts on the convergence rate of PSO, which is calculated adaptively as *w*=(max_iteration − current_iteration/(2 *∗* max_iteration))+0.4 (max_interation is the maximum number of iterations in the algorithm and current_interation is the number of current iterations), *c*_1_ and *c*_2_ are the positive constants, and *r*_1_ and *r*_2_ are uniformly distributed random numbers in [0, 1].

With the updated velocities, each particle changes its position according to the following equation:

(6) $$\begin{array}{c} x_{i}\left(t+1\right)=x_{i}\left(t\right)+v_{i}\left(t+1\right). \end{array}$$

## 3. Methods

### 3.1. Restricted Gene Expression Programming

**Figure 2:**
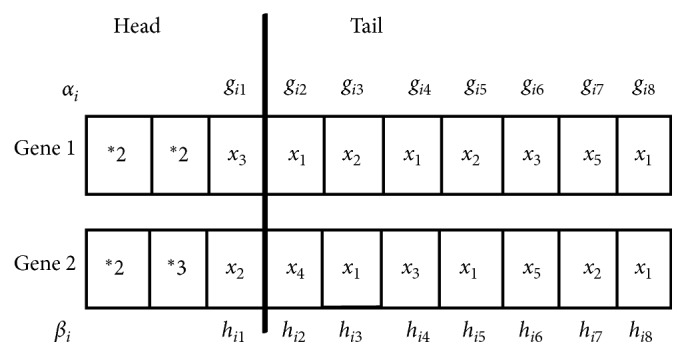
The phenotype of chromosome in RGEP with parameters.

The restricted gene expression programming (RGEP) as the improved version of GEP was proposed to identify the S-system model for gene regulatory network (GRN) inference [37]. The flowchart of RGEP is described as follows:

1. Initialize the population. One example of chromosome in population is depicted in Figure 2. Each chromosome contains two genes and each gene contains head part and tail part, which are created randomly using the function set (*F*) and variable set (*T*):

(7) $$\begin{array}{c} \left\{\begin{array}{c} F=\left\{*1,*2,*3,\ldots ,*n\right\},\\ T=\left\{x_{1},x_{2},\ldots ,x_{m},R\right\}, \end{array}\right. \end{array}$$

where *∗n* is an operation of *n* variables multiplying, *x*_*i*_ is the variable, *m* is the number of input variables, and *R* is the constant.

**Figure 3:**
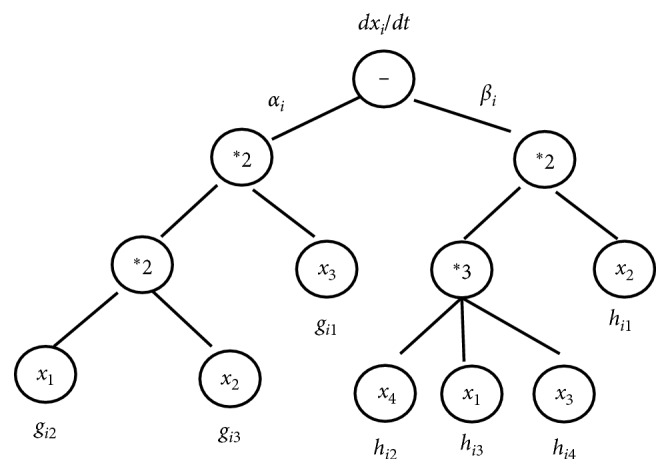
The expression tree of chromosome in RGEP with parameters.

In order to make the chromosome similar to the S-system, each gene is allocated the corresponding parameters. For gene 1, *α*_*i*_ is given as its coefficient and each variable is given exponent *g*_*ij*_. For gene 2, *β*_*i*_ is given as its coefficient and each variable is given exponent *h*_*ij*_. Two genes are connected by the subtraction operation (−).  Figure 3 shows the expression tree (ET) of Figure 2, and its corresponding S-system model is expressed as follows:

(8) $$\begin{array}{c} \frac{dx_{i}}{dt}=\alpha_{i}x_{3}^{g_{i1}}x_{1}^{g_{i2}}x_{2}^{g_{i3}}-\beta_{i}x_{2}^{h_{i1}}x_{4}^{h_{i2}}x_{1}^{h_{i3}}x_{3}^{h_{i4}}. \end{array}$$

(2) According to the given fitness function, evaluate the population with the training samples. In this process, the S-system model is solved by the fourth-order Runge–Kutta method [38]. For the differential equation (*dy*/*dt*)=*f*(*x*, *y*), the solution is as follows:

(9) $$\begin{array}{c} k_{1}=f\left(x\left(t\right),y\left(t\right)\right),\\ k_{2}=f\left(x\left(t\right)+\frac{h}{2},y\left(t\right)+h\;*\;\frac{k_{1}}{2}\right),\\ k_{3}=f\left(x\left(t\right)+\frac{h}{2},y\left(t\right)+h\;*\;\frac{k_{2}}{2}\right),\\ k_{4}=f\left(x\left(t\right)+h,y\left(t\right)+h\;*\;k_{3}\right),\\ y\left(t+1\right)=y\left(t\right)+h\;*\;\frac{k_{1}+2k_{2}+2k_{3}+k_{4}}{6}, \end{array}$$

where *h* is the step size.

(3) If the optimal solution appears, RGEP is terminated; otherwise, turn to (4).
(4) Selection, recombination, and mutation are used for reproduction of each chromosome, which are introduced in Reference [37].

In the initial stage of structural optimization, the symbols of the chromosome in RGEP are randomly selected, including function symbols and variable symbols. With training data, reproduction operators are used to optimize and change the chromosomal symbols in the optimization process. The optimized S-system structure does not contain all the input variables. According to the training data, RGEP could automatically select the appropriate input variables. In Figure 2, we can find that the coefficients *α*_*i*_ and *β*_*i*_ and the exponents *g*_*i*1_, *g*_*i*2_, *g*_*i*3_, *h*_*i*1_, *h*_*i*2_, *h*_*i*3_, and  *h*_*i*4_ are needed to be optimized. In this paper, the parameters in each chromosome are optimized by a hybrid intelligent algorithm based on BSO algorithm and PSO algorithm.

### 3.2. Hybrid Optimization Algorithm

**Figure 4:**
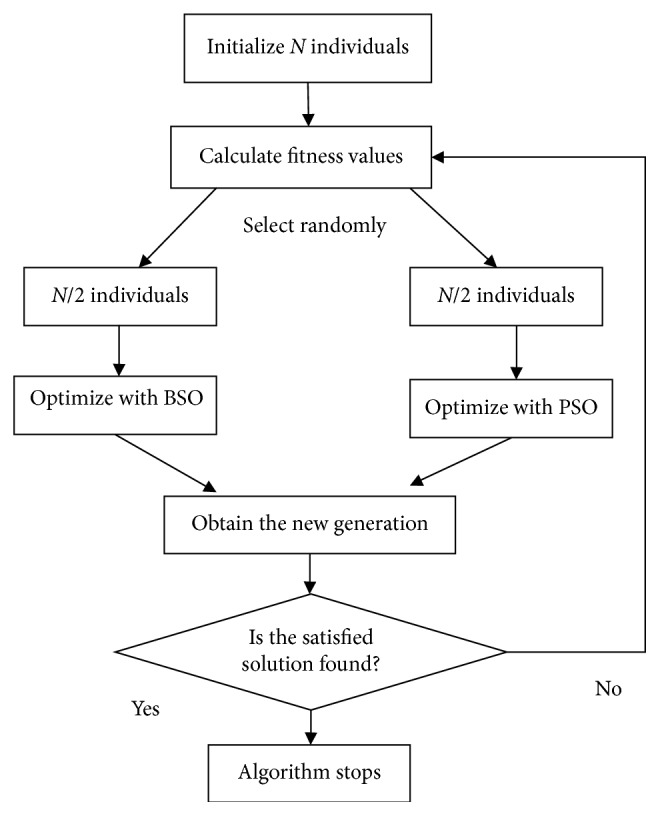
The flowchart of BSO-PSO algorithm.

The BSO algorithm is suitable for solving the problem of multipeak and high-dimensional function. The PSO algorithm has the advantages of easy realization, high accuracy, and fast convergence. But these two methods are easy to converge prematurely and fall into local optimum. In order to improve the diversity of population, a novel hybrid intelligent algorithm based on BSO and PSO (BSO-PSO) is proposed. In the BSO-PSO algorithm, the half of individuals are selected randomly and optimized by BSO. And the other individuals are optimized by PSO. The flowchart is described in Figure 4.

### 3.3. Time Series Data Forecasting Using S-System

**Figure 5:**
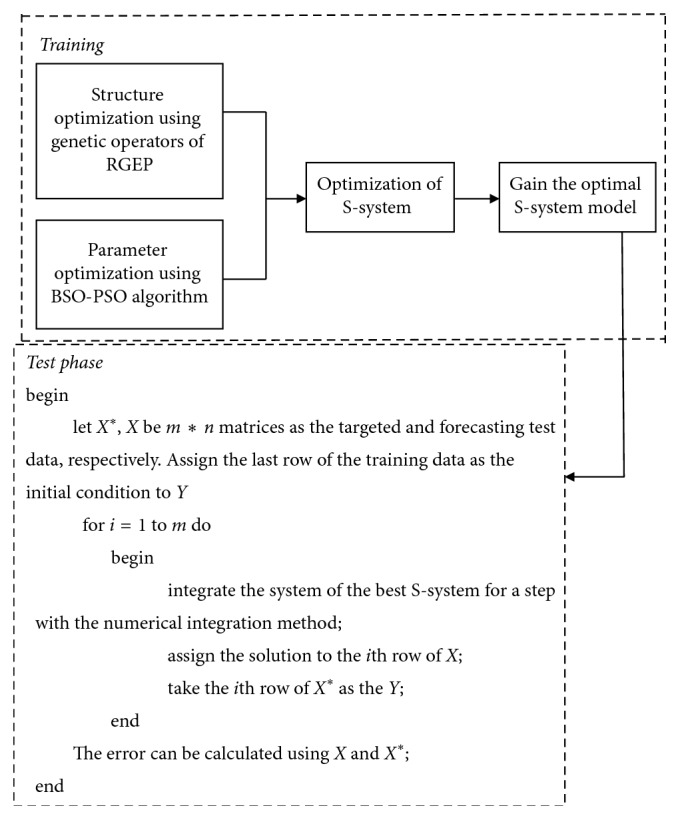
The flowchart of time series data forecasting using S-system.

The flowchart of time series forecasting using the S-system model is described in Figure 5. During the training phase, the S-system model is optimized according to the genetic operators of RGEP, hybrid intelligent algorithm, and training dataset. During the test phase, the optimal S-system is used to make the prediction of the stock index. The detailed process is described as follows.

#### 3.3.1. Training Phase

1. Initialize the S-system population with the structure and parameters. Each S-system is encoded as the RGEP chromosome, which is described in Figure 2.
2. With the training samples, the S-system is solved by equation (4) and the fitness value of each S-system is calculated. Search the best S-system according to the fitness values. If the optimal model is found, the algorithm stops.
3. Selection, recombination, and mutation are used to search the optimal structure of the S-system. Go to step (2).
4. At some iterations in RGEP, BSO-PSO algorithm is used to optimize the parameters of RGEP chromosomes. In this process, the structure of the S-system model is fixed. According to the structure of the model, the number of parameters (*α*_*i*_, *β*_*i*_, *g*_*ij*_, and  *h*_*ij*_) is counted. With the hybrid intelligent algorithm, search and update the optimal parameters of each S-system.

#### 3.3.2. Testing Phase

With the data at the previous time point, the optimal S-system model obtained in the training phase is solved and the data at the current time point are predicted. Repeat this procedure until that the data at all testing time points have been predicted. According to the predicted data and target data, the predicted error is calculated.

## 4. Results and Discussion

### 4.1. Data and Evaluation Standard

Five stock indexes such as Dow Jones Index (DJI), Hang Seng Index (HSI), NASDAQ Index (NASI), SSE (Shanghai Stock Exchange) Composite Index (SSEI), and SZSE Component Index (SZSEI) are proposed to test the performance of our method. Seventy percent of the data are used for training, and 30% of the data are used for testing. The descriptions of five stock indexes are listed in Table 1.

RMSE (root mean square error), MAP (mean absolute percentage), and MAPE (mean absolute percentage error), *R*^2^ (coefficient of multiple determinations for multiple regressions), ARV (average relative variance), and VAF (variance accounted for) are proposed to evaluate the performance of our method [30, 39]:

(10) $$\begin{array}{c} \text{RMSE}=\sqrt{\frac{1}{N}\overset{N}{\underset{i=1}{\sum }}{\left(f_{\text{target}}^{i}-f_{\text{forecast}}^{i}\right)}^{2}},\\ \text{MAP}=\max\left(\frac{\left|f_{\text{target}}^{i}-f_{\text{forecast}}^{i}\right|}{f_{\text{forecast}}^{i}}\times 100\right),\\ \text{MAPE}=\frac{1}{N}\overset{N}{\underset{i=1}{\sum }}\left(\frac{\left|f_{\text{target}}^{i}-f_{\text{forecast}}^{i}\right|}{f_{\text{forecast}}^{i}}\right)\times 100,\\ R^{2}=1-\frac{\sum_{i=1}^{N}{\left(f_{\text{target}}^{i}-f_{\text{forecast}}^{i}\right)}^{2}}{\sum_{i=1}^{N}{\left(f_{\text{target}}^{i}-\bar{f}\right)}^{2}},\\ \text{ARV}=\frac{\sum_{i=1}^{N}{\left(f_{\text{target}}^{i}-f_{\text{forecast}}^{i}\right)}^{2}}{\sum_{i=1}^{N}{\left(f_{\text{forecast}}^{i}-\bar{f}\right)}^{2}},\\ \text{VAF}=\left(1-\frac{\sum_{i=1}^{N}{\left(f_{\text{target}}^{i}-y_{\text{forecast}}^{i}\right)}^{2}}{\sum_{i=1}^{N}{\left(f_{\text{target}}^{i}\right)}^{2}}\right)\times 100\%, \end{array}$$

where *N* is the number of stock sample points, *f*_target_^*i*^ is the real stock value at the *i*th time point, *f*_forecast_^*i*^ is the predicting stock value at the *i*th time point, and $\bar{f}$ is the mean of stock indexes.

### 4.2. Prediction Results

In order to test the performance of our method clearly, five states of the art methods (Deep Recurrent Neural Network (DRNN) [40], FNT [19], RBFNN [17], BPNN [14], and ARIMA [8]) are also used to predict five stock indexes.

**Figure 6:**
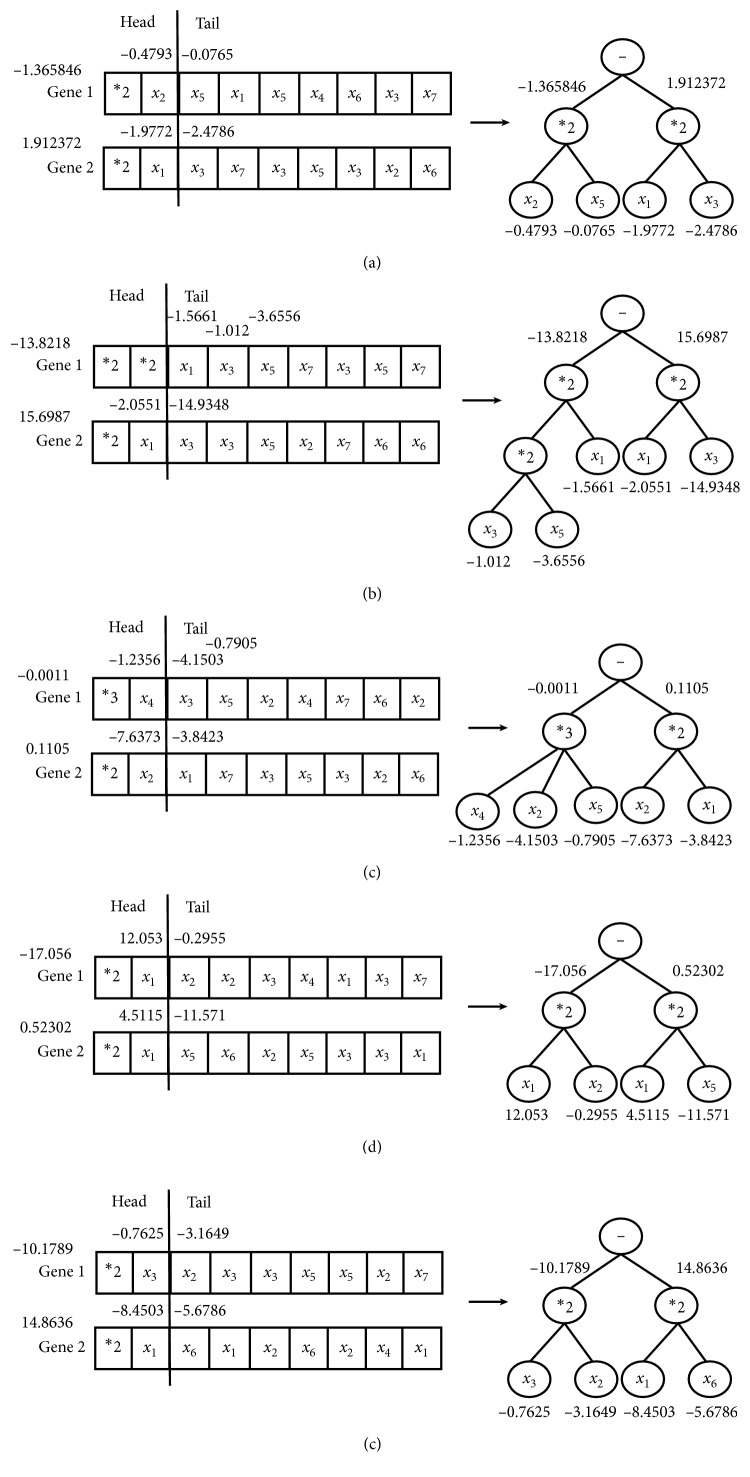
The optimal phenotypes and expression trees for a-week-ahead prediction with five stock indexes: DJI (a), HIS (b), NASI (c), SSEI (d), and SZSEI (e).

**Figure 7:**
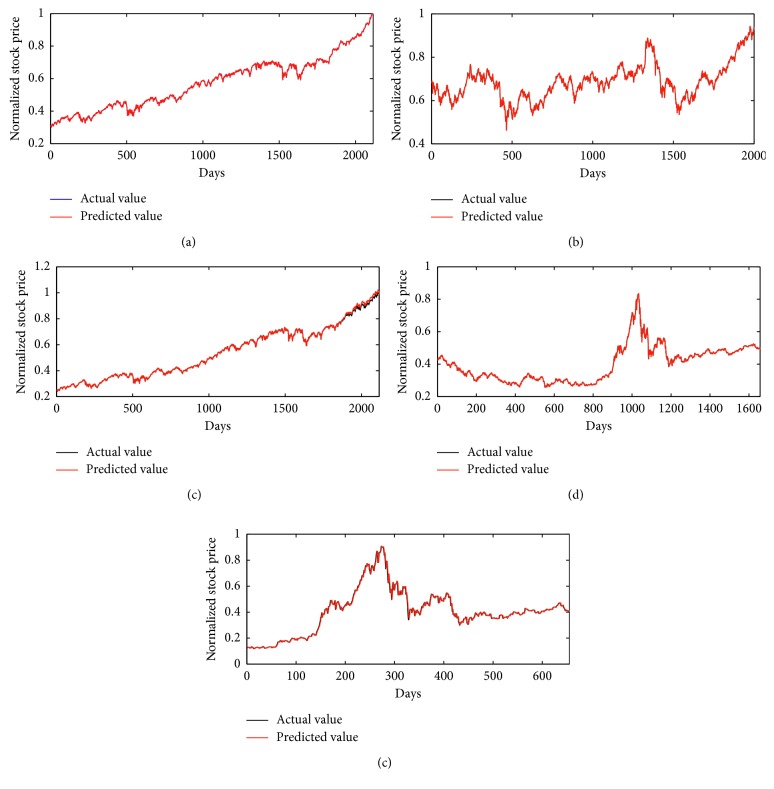
The prediction and actual results for a-week-ahead prediction with five stock indexes: DJI (a), HIS (b), NASI (c), SSEI (d), and SZSEI (e).

For 1-week-ahead prediction problem, function set is *F*={*∗*1, *∗*2, *∗*3, *∗*4} and variable set is *T*={*x*_1_, *x*_2_,…, *x*_7_} in the RGEP method. By optimizing S-system models by our method, we could obtain the optimal phenotypes and expression trees (ETs) with five stock indexes, which are described in Figure 6. Five optimal S-system models gained are listed in Table 2 for five stock datasets. The forecasting results of five stock indexes by our method are depicted in Figure 7. From Figure 7, it can be clearly seen that the predicting curves are very near to the target ones, and the errors are nearly zero.

Comparison results of different prediction models' performance on five stock indexes are listed in Table 3. From Table 3, among the past five states of the art methods, the DRNN model performs best for five stock indexes prediction. But in terms of six indicators (RMSE, MAP, ARV, MAPE, *R*^2^, and VAF), our proposed method has better performance than the DRNN model. In terms of RMSE, our method is 34.8% lower than DRNN for DJI dataset, 46.4% lower than DRNN for HSI dataset, 40.4% lower than DRNN for NASI dataset, 19.8% lower than DRNN for SSEI dataset, and 7.4% lower than DRNN for SZSEI dataset. In terms of ARV, our method is 58.7% lower than DRNN for DJI dataset, 67.1% lower than DRNN for HSI dataset, 68.8% lower than DRNN for NASI dataset, 36.9% lower than DRNN for SSEI dataset, and 16.5% lower than DRNN for SZSEI dataset. In terms of MAPE, our method is 37.5% lower than DRNN for DJI dataset, 48% lower than DRNN for HSI dataset, 42.9% lower than DRNN for NASI dataset, 35.2% lower than DRNN for SSEI dataset, and 18% lower than DRNN for SZSEI dataset. In terms of VAF, our method is closer to 100% than DRNN for five stock indexes. It could be seen clearly that our proposed method could improve the prediction accuracy sharply.

**Figure 8:**
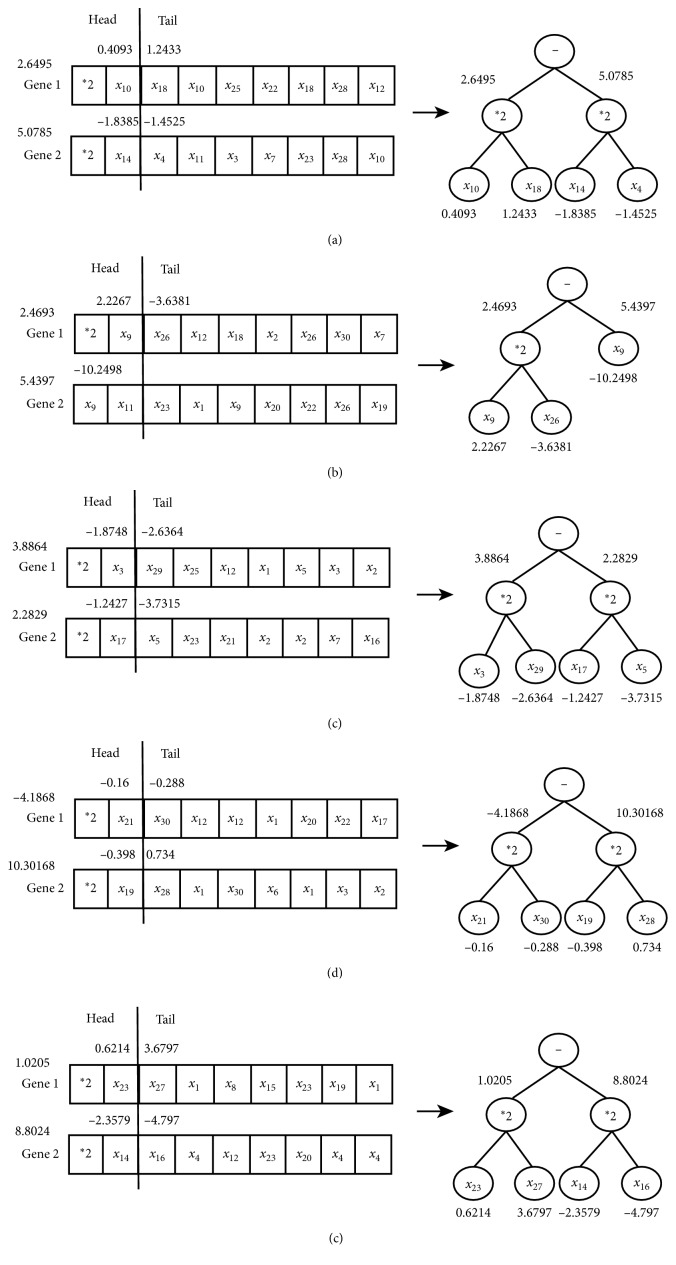
The optimal phenotypes and expression trees for a-month-ahead prediction with five stock indexes: DJI (a), HIS (b), NASI (c), SSEI (d), and SZSEI (e).

**Figure 9:**
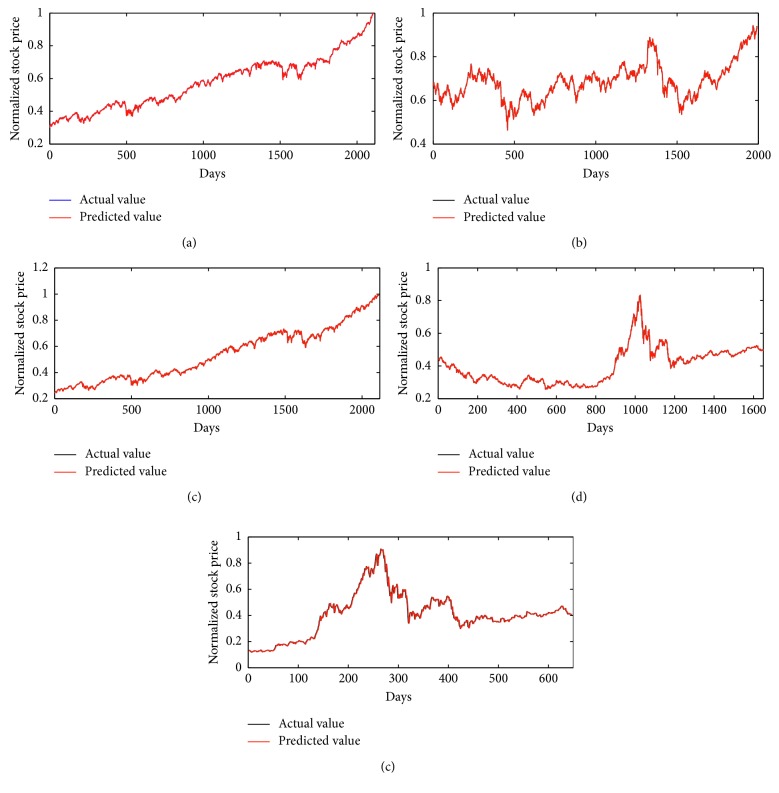
The prediction and actual results for a-month-ahead prediction with five stock indexes: DJI (a), HIS (b), NASI (c), SSEI (d), and SZSEI (e).

For 1-month-ahead prediction problem, function set is *F*={*∗*1, *∗*2, *∗*3, *∗*4} and variable set is *T*={*x*_1_, *x*_2_,…, *x*_30_} in the RGEP method. With five stock indexes, we obtain five optimal phenotypes and expression trees (ETs), which are described in Figure 8. According to five ETs, the S-system models gained are listed in Table 4. The forecasting results of five stock indexes by our method are depicted in Figure 9. From Figure 9, we could see clearly that the predicting and target curves are very close.

Six prediction models are used to forecast five stock indexes, and the prediction results are listed in Table 5. From Table 5, it can be seen that the five indicators (RMSE, ARV, MAPE, *R*^2^, and VAF) of our method are all the best of these six methods with the three datasets (DJI, HIS, and NASI). The DRNN model has the highest MAP, which are 2.1368, 2.9568, and 6.3901, respectively. For SSEI and SZSEI datasets, our proposed method has the best performance in terms of RMSE, MAP, ARV, MAPE, *R*^2^, and VAF. In terms of ARV, our method is closer to 0 than other five methods. In terms of *R*^2^, our method is closer to 1. In terms of VAF, our method is closer to 100%. Thus, our proposed forecasting model tends to be more accurate.

### 4.3. Hybrid Intelligent Algorithm Analysis

In order to test the performance of our proposed hybrid intelligent algorithm, we use BSO and PSO to optimize the parameters of S-system models in the comparison experiments. Through 20 runs, with DJI dataset, the a-week-ahead prediction results by three evolutionary methods are listed in Table 6, which contains the best value, worse value, mean value, and standard error (SD) of the mean of 20-run RMSEs. From Table 6, we can see that through 20 runs, the best RMSE values by three evolutionary methods are very close, but the other three indicators seem to have a big difference. Our hybrid intelligent algorithm could obtain smaller worse RMSE, mean RMSE, and SD than PSO and BSO, which indicates that our hybrid intelligent algorithm is more robust and not easier to fall into local optimum than PSO and BSO.

**Figure 10:**
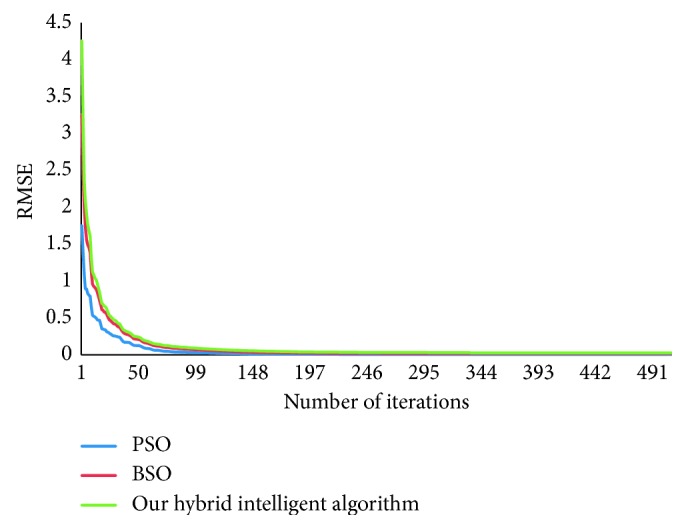
Comparison of error convergence characteristics of our hybrid intelligent algorithm, BSO, and PSO for a-week-ahead prediction using DJI dataset.

Figure 10 depicts the comparison of the RMSE convergence rate obtained from the application of our hybrid intelligent algorithm, BSO and PSO with DJI dataset for a-week-ahead prediction. Figure 10 reveals that our proposed intelligent algorithm has faster convergence than PSO and BSO in the early stage of the optimization process. When the number of iterations reaches 200, the RMSE convergence rate is dropping to 10^−3^ that indicates the significant minimization of error.

### 4.4. Restricted Gene Expression Programming Analysis

**Figure 11:**
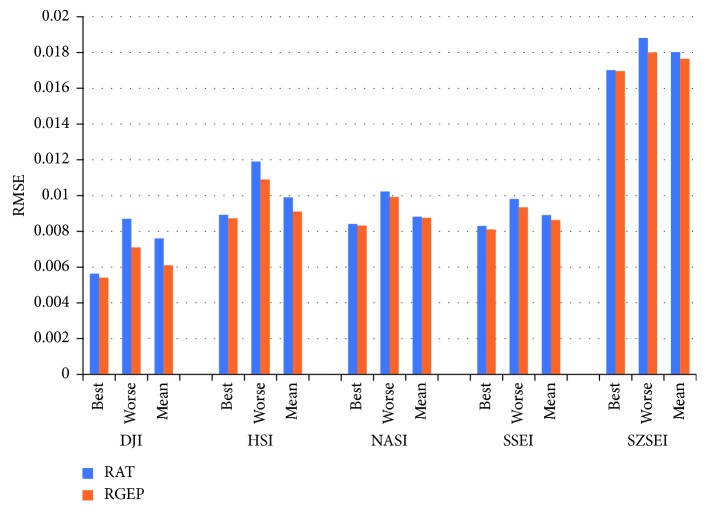
Prediction comparison of two optimization algorithms for a-week-ahead prediction with five stock indexes.

In order to test the performance of restricted gene expression programming for S-system optimization, the restricted additive tree is used to optimize the structure of the S-system model in the comparison experiments. Through 20 runs, with five stock indexes, the a-week-ahead prediction results by RGEP and RAT are depicted in Figure 11, which contains the best values, worse values, and mean values of 20-run RMSEs. From Figure 11, it could be clearly seen that RGEP could obtain smaller best, worse, and mean RMSE values than RAT, which reveal that RGEP could search the optimal S-system model more easily than RAT.

## 5. Conclusions

In this paper, a novel stock prediction method based on the S-system model is proposed to forecast the stock market. An improved gene expression programming (RGEP) is proposed to represent and optimize the structure of the S-system model. A hybrid intelligent algorithm based on BSO and PSO is used to optimize the parameters of the S-system model. Our proposed method is tested by predicting five real stock price datasets such as DJI, HIS, NASI, SSEI, and SZSEI. The results of predicting the stock price a-week-ahead and a-month-ahead reveal that our method could predict the stock index accurately and performs better than DRNN, FNT, RBFNN, BPNN, and ARIMA.

The convincing performance of our method is mainly due to three aspects. The first is that the nonlinear ordinary differential equation model S-system has strong nonlinear modeling and forecasting ability. Table 6 and Figure 10 show that our hybrid intelligent algorithm is more robust and not easier to fall into local optimum than PSO and BSO. From Tables 2 and 4, we can see that the optimal S-system models contain a portion of input variables. This is because our method can automatically select the proper input variables according to different stock data, which also prevents overfitting problem.

## Figures and Tables

**Table 1:** Parameters of five stock indexes.

Parameters	DJI	HSI	NASI	SSEI	SZSEI
Time interval	1/2/1990–12/29/2017	1/2/1991–12/29/2017	1/2/1990–12/29/2017	1/1/1996–12/29/2017	1/2/2008–12/30/2016
Train data for week-ahead prediction	4936	4666	4936	3866	1528
Test data for week-ahead prediction	2115	2000	2115	1657	655
Train data for month-ahead prediction	4918	4649	4918	3849	1511
Test data for month-ahead prediction	2108	1992	2108	1649	647

**Table 2:** Optimal S-system models of five stock datasets for a-week-ahead prediction.

Type of datasets	Optimal S-system model
DJI	$\dot{f}=-1.365846x_{2}^{-0.4793}x_{5}^{-0.0765}-1.912372x_{1}^{-1.9772}x_{3}^{-2.4786}$
HSI	$\dot{f}=-13.8218x_{1}^{-1.5661}x_{3}^{-1.012}x_{5}^{-3.6556}-15.6987x_{1}^{-2.0551}x_{3}^{-14.9348}$
NASI	$\dot{f}=-0.0011x_{4}^{-1.2356}x_{3}^{-4.1503}x_{5}^{-0.7905}-0.1105x_{2}^{-7.6373}x_{1}^{-3.8423}$
SSEI	$\dot{f}=-17.056x_{1}^{12.053}x_{2}^{-0.2955}-0.52302x_{1}^{4.5115}x_{5}^{-11.571}$
SZS	$\dot{f}=-10.1789x_{3}^{-0.7625}x_{2}^{-3.1649}-14.8636x_{1}^{-8.4503}x_{6}^{-5.6786}$

**Table 3:** Comparison results of six methods for a-week-ahead prediction.

Stock index	Method	RMSE	MAP	ARV	MAPE	*R* ^2^	VAF (%)
DJI	Our method	**0.005411**	**6.9911**	**0.00113**	**0.73146**	**0.99887**	**99.992**
	DRNN	0.0083	8.4909	0.002735	1.1696	0.99726	99.981
	FNT	0.015427	14.907	0.00613	1.8023	0.98939	99.933
	RBFNN	0.016188	24.473	0.01057	2.2631	0.98943	99.927
	BPNN	0.049026	22.721	0.060301	5.5923	0.9397	99.328
	ARIMA	0.052472	20.924	0.071326	5.9773	0.92867	99.230

HSI	Our method	**0.008725**	**6.8824**	**0.01116**	**0.97359**	**0.98883**	**99.984**
	DRNN	0.016272	13.384	0.033924	1.8718	0.96608	99.944
	FNT	0.020128	19.261	0.065331	2.2759	0.93467	99.915
	RBFNN	0.023406	25.32	0.072545	2.7067	0.92756	99.885
	BPNN	0.035987	44.738	0.12867	4.1357	0.87133	99.729
	ARIMA	0.013361	13.817	0.026688	1.5367	0.97331	99.963

NASI	Our method	**0.008324**	**7.9168**	**0.001707**	**1.0035**	**0.99829**	**99.979**
	DRNN	0.013969	11.069	0.005465	1.757	0.99453	99.941
	FNT	0.016468	32.352	0.006859	2.5336	0.99314	99.918
	RBFNN	0.03669	37.371	0.027513	4.5327	0.97249	99.591
	BPNN	0.046	17.5	0.042533	5.973	0.95747	99.353
	ARIMA	0.049849	18.46	0.093189	5.31	0.90681	99.245

SSEI	Our method	**0.008105**	**9.957**	**0.00535**	**1.1271**	**0.99465**	**99.962**
	DRNN	0.010107	12.959	0.008481	1.7396	0.99152	99.941
	FNT	0.014559	18.931	0.018903	2.2848	0.9811	99.878
	RBFNN	0.014681	20.06	0.018024	2.1804	0.98198	99.876
	BPNN	0.035922	32.768	0.091613	6.9046	0.90839	99.256
	ARIMA	0.020766	20.533	0.029814	3.9766	0.97019	99.752

SZSEI	Our method	**0.016959**	**16.079**	**0.009762**	**2.4933**	**0.99024**	**99.851**
	DRNN	0.018315	19.783	0.011685	3.0419	0.98831	99.826
	FNT	0.018571	21.67	0.012189	3.0233	0.98781	99.821
	RBFNN	0.023881	31.222	0.018031	3.8187	0.98197	99.704
	BPNN	0.027297	41.441	0.027768	4.0751	0.97223	99.614
	ARIMA	0.029022	26.983	0.02844	4.8583	0.97156	99.563

**Table 4:** Optimal S-system models of five stock datasets for a-month-ahead prediction.

Type of datasets	Optimal S-system model
DJI	$\dot{f}=2.6495x_{10}^{0.4093}x_{18}^{1.2433}-5.0785x_{14}^{-1.8385}x_{4}^{-1.4525}$
HSI	$\dot{f}=2.4693x_{9}^{2.2267}x_{26}^{-3.6381}-5.4397x_{9}^{-10.2498}$
NASI	$\dot{f}=3.8864x_{3}^{1.8748}x_{29}^{-2.6364}-2.2829x_{17}^{-1.2427}x_{5}^{-3.7315}$
SSEI	$\dot{f}=-4.1868x_{21}^{-0.16}x_{30}^{0.288}-10.30168x_{19}^{-0.398}x_{28}^{0.734}$
SZSEI	$\dot{f}=1.0205x_{23}^{0.6214}x_{27}^{3.6797}-8.8024x_{14}^{-2.3579}x_{16}^{-4.797}$

**Table 5:** Comparison results of six methods for a-month-ahead prediction.

Stock index	Method	RMSE	MAP	ARV	MAPE	*R* ^2^	VAF (%)
DJI	Our method	**0.005413**	6.9911	**0.001139**	**0.73002**	**0.99886**	**99.992**
	DRNN	0.007741	**2.1368**	0.002616	1.4501	0.99738	99.983
	FNT	0.012504	2.4418	0.007481	1.9062	0.99252	99.956
	RBFNN	0.013379	3.9361	0.007573	2.5368	0.99243	99.950
	BPNN	0.048029	10.1	0.13547	7.3864	0.86453	99.356
	ARIMA	0.052385	93.126	0.11662	7.3731	0.88338	99.234

HSI	Our method	**0.008645**	6.95	**0.010944**	**0.96689**	**0.98906**	**99.984**
	DRNN	0.011502	**2.9568**	0.027542	1.489	0.97246	99.972
	FNT	0.014388	4.3212	0.046465	1.7348	0.95353	99.957
	RBFNN	0.044134	35.289	0.41237	5.1249	0.58763	99.592
	BPNN	0.045971	55.247	0.22748	5.3369	0.77252	99.557
	ARIMA	0.061245	53.144	0.54399	7.0813	0.45601	99.214

NASI	Our method	**0.0057**	7.9168	**8.37E-04**	**0.83204**	**0.99916**	**99.990**
	DRNN	0.031166	**6.3901**	0.031964	5.3044	0.96804	99.706
	FNT	0.031894	9.3407	0.037088	3.7881	0.96291	99.692
	RBFNN	0.035863	11.232	0.04911	3.9605	0.95089	99.610
	BPNN	0.047487	9.9709	0.083254	7.5185	0.91675	99.317
	ARIMA	0.098081	91.02	0.3589	12.177	0.6411	97.086

SSEI	Our method	**0.003073**	**1.669**	**8.14E-04**	**0.61418**	**0.99919**	**99.995**
	DRNN	0.008104	9.957	0.005335	1.1292	0.99467	99.962
	FNT	0.033005	34.807	0.070553	5.5303	0.92945	99.372
	RBFNN	0.04973	76.098	0.12737	8.4546	0.87263	98.574
	BPNN	0.053661	111.48	0.14219	9.4878	0.85781	98.340
	ARIMA	0.071626	87.753	0.2008	13.551	0.7992	97.043

SZSEI	Our method	**0.017003**	**16.007**	**0.010063**	**2.4916**	**0.98994**	**99.852**
	DRNN	0.045067	19.483	0.13092	7.3945	0.88439	98.959
	FNT	0.06323	25.729	0.2267	11.628	0.7733	97.950
	RBFNN	0.071342	35.295	0.32104	11.772	0.67896	97.390
	BPNN	0.082818	37.58	0.51956	16.103	0.48044	96.483
	ARIMA	0.084487	117.1	0.32894	12.521	0.67106	96.340

**Table 6:** The averaged RMSE results of three evolutionary methods for a-week-ahead prediction.

Method	Best	Worse	Mean	SD
Our hybrid intelligent algorithm	0.005411	0.0071	0.0061	0.00065
BSO	0.005608	0.0085	0.0072	0.00074
PSO	0.005475	0.0098	0.0079	0.00081

## Data Availability

The five stock indexes could be downloaded freely at https://hk.finance.yahoo.com/.
